# Host Resistance to Plasmodium-Induced Acute Immune Pathology Is Regulated by Interleukin-10 Receptor Signaling

**DOI:** 10.1128/IAI.00941-16

**Published:** 2017-05-23

**Authors:** Carla Claser, J. Brian De Souza, Samuel G. Thorburn, Georges Emile Grau, Eleanor M. Riley, Laurent Rénia, Julius C. R. Hafalla

**Affiliations:** a Laboratory of Pathogen Immunobiology, Singapore Immunology Network (SIgN), Agency for Science, Technology and Research (A*STAR), Biopolis, Singapore; b Division of Infection and Immunity, University College London Medical School, London, United Kingdom; c Immunology and Infection Department, Faculty of Infectious and Tropical Diseases, London School of Hygiene and Tropical Medicine, London, United Kingdom; d Vascular Immunology Unit, Department of Pathology, School of Medical Sciences, Bosch Institute, The University of Sydney, Camperdown, New South Wales, Australia; University of South Florida

**Keywords:** malaria, Plasmodium berghei, T cells, IL-10, IFN-γ, immunopathology

## Abstract

The resolution of malaria infection is dependent on a balance between proinflammatory and regulatory immune responses. While early effector T cell responses are required for limiting parasitemia, these responses need to be switched off by regulatory mechanisms in a timely manner to avoid immune-mediated tissue damage. Interleukin-10 receptor (IL-10R) signaling is considered to be a vital component of regulatory responses, although its role in host resistance to severe immune pathology during acute malaria infections is not fully understood. In this study, we have determined the contribution of IL-10R signaling to the regulation of immune responses during Plasmodium berghei ANKA-induced experimental cerebral malaria (ECM). We show that antibody-mediated blockade of the IL-10R during P. berghei ANKA infection in ECM-resistant BALB/c mice leads to amplified T cell activation, higher serum gamma interferon (IFN-γ) concentrations, enhanced intravascular accumulation of both parasitized red blood cells and CD8^+^ T cells to the brain, and an increased incidence of ECM. Importantly, the pathogenic effects of IL-10R blockade during P. berghei ANKA infection were reversible by depletion of T cells and neutralization of IFN-γ. Our findings underscore the importance of IL-10R signaling in preventing T-cell- and cytokine-mediated pathology during potentially lethal malaria infections.

## INTRODUCTION

The regulation of pathological mechanisms leading to cerebral malaria (CM), a severe complication of infection with the malaria parasite Plasmodium falciparum, remains obscure. Experimental CM (ECM) due to Plasmodium berghei ANKA infection in susceptible C57BL/6 mice mimics the neurological signs observed during human CM, including ataxia and/or paralysis, which rapidly deteriorate to convulsions, coma, and death 7 to 10 days postinfection (1, 2). Histological examination of both CM and ECM brain sections reveals the presence of petechial hemorrhages (3–5). Furthermore, both CM and ECM are characterized by the accumulation of parasitized red blood cells (pRBCs) and leukocytes in the cerebral microvasculature.

In C57BL/6 mice, the development of ECM is associated with CD8α^+^ Clec9A^+^ dendritic cells (DCs), which prime naive CD4^+^ and CD8^+^ T cells to become effector cells and secrete proinflammatory cytokines such as gamma interferon (IFN-γ) (6, 7). The production of IFN-γ by CD4^+^ T cells is thought to enhance the recruitment of effector CD8^+^ T cells to brain microvessels, where pRBCs also accumulate (8, 9). These effector CD8^+^ T cells, upon recognition of the parasite-derived epitopes presented by the brain endothelial cells (10, 11), secrete perforin and granzymes, leading to breaching of the blood-brain barrier (12–14) and causing hemorrhages. Besides neurological impairment, P. berghei ANKA-infected C57BL/6 mice develop a multiorgan disease, and in the absence of cerebral pathology, animals die at a later time point because of anemia and hyperparasitemia (9).

In contrast, P. berghei ANKA infection of BALB/c mice does not generally lead to ECM and therefore this strain is considered ECM resistant, although the infected animals succumb to anemia and hyperparasitemia 2 to 3 weeks postinfection (1, 15). However, the immune mechanisms that confer resistance to ECM remain poorly understood. We previously showed that T cell inhibitory pathways, cytotoxic T lymphocyte antigen 4 (CTLA-4, CD152), and programmed death 1 (PD-1, CD279)/PD ligand 1 (PD-L1, CD274) independently regulate host resistance to ECM (15). Blockade of the CTLA-4 or PD-1/PD-L1 pathway in P. berghei ANKA-infected BALB/c mice led to the development of ECM with characteristics similar to those observed in C57BL/6 mice.

Interleukin (IL-10), an anti-inflammatory cytokine, is a principal regulator of immunity to infection. IL-10 signaling through its receptor (IL-10R, CD210) is known to attenuate the production of IFN-γ and other proinflammatory responses (16, 17), which may otherwise induce immune pathology during acute infections. In the nonlethal models of P. chabaudi and P. yoelii blood stage malaria infection, deficiency in IL-10 signaling is associated with increased IFN-γ secretion and good parasite control at the expense of exacerbated immune pathology (18–20). Likewise, IL-10 deficiency is fatal in the avirulent murine models of both Toxoplasma gondii and Trypanosoma cruzi (21, 22). Together, these studies clearly indicate a critical role for the IL-10R signaling pathway in preventing pathology. IL-10R signaling attenuates the production of IFN-γ and other proinflammatory responses responsible for inducing immune-mediated pathology during acute parasitic infections.

In the present study, we hypothesized that IL-10R signaling also regulates T-cell-mediated inflammatory responses in ECM-resistant BALB/c mice, thereby preventing the onset of ECM. Blockade of the IL-10R during P. berghei ANKA infection of BALB/c mice results in acute immune-mediated pathology with features resembling those of ECM in susceptible mice. Therefore, the IL-10R signaling pathway appears to effectively maintain the equilibrium between pathogen clearance and tissue damage during the early stages of a lethal malaria infection in BALB/c mice.

## RESULTS

### Blockade of IL-10R signaling induces ECM in normally resistant BALB/c mice.

**FIG 1:**
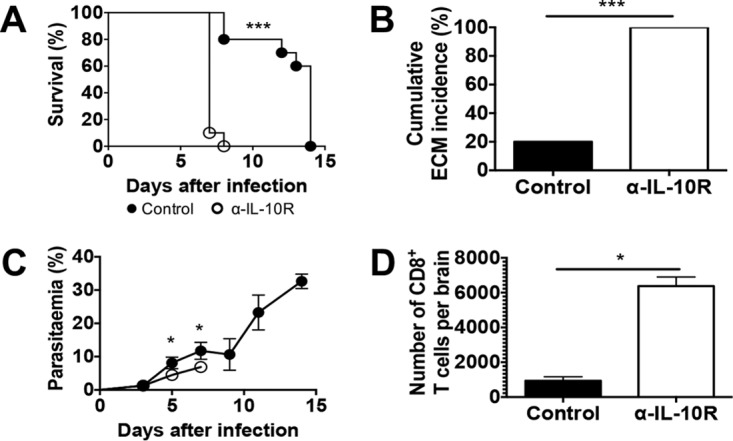
IL-10R blockade in P. berghei ANKA-infected BALB/c mice results in ECM. BALB/c mice were infected i.v. with 10^4^ P. berghei ANKA pRBCs and treated with anti-IL-10R antibodies. Control mice received either no antibody or rat IgG. (A) Cumulative survival curve. Closed circles (●), control (*n* = 12); open circles (○), anti-IL-10R antibody (α-IL-10R) (*n* = 12). ***, *P* < 0.0001 (log rank [Mantel-Cox] test). (B) Cumulative incidence of mice developing ECM (based on neurological signs, i.e., ataxia and paralysis). ***, *P* < 0.0001 (Fisher's exact test). Mice that survived were euthanized on day 14 because of high parasitemia and anemia. (C) Parasitemia levels, shown as the mean ± the standard deviation, of P. berghei ANKA-infected mice. Closed circles, control; open circles, anti-IL-10R antibody. The data shown are representative of three independent experiments with four to six mice per group. *, *P* < 0.05 (Mann-Whitney U test). (D) Absolute numbers of CD8^+^ T lymphocytes that have accumulated in the brain. The data shown (mean ± standard deviation) are representative of two independent experiments with four mice per group. *, *P* < 0.05 (Mann-Whitney U test).

To establish whether IL-10R signaling regulates ECM pathogenesis in an otherwise ECM-resistant mouse strain, the outcomes of P. berghei ANKA infection in control mice and mice treated with blocking antibodies to IL-10R were compared. While control BALB/c mice (treated with rat IgG or phosphate-buffered saline [PBS]) survived for up to 2 weeks postinfection, mice treated with anti-IL-10R antibody developed classical neurological signs of ECM and were euthanized on day 7 or 8 postinfection (Fig. 1A and B). Both the survival curve and the cumulative ECM incidence of anti-IL-10R antibody-treated mice differ significantly from those of control mice. Strikingly, anti-IL-10R antibody-treated mice presented significantly lower parasitemia levels on days 5 and 7 postinfection than control mice (Fig. 1C). Consistent with the development of ECM, the number of accumulated intravascular CD8^+^ T cells was higher in the brains of anti-IL-10R antibody-treated mice than in those of control mice (Fig. 1D).

**FIG 2:**
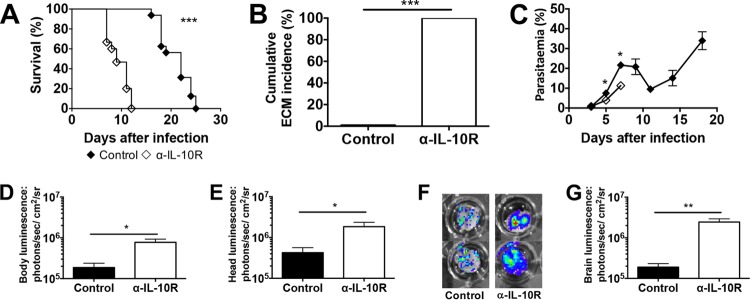
ECM after IL-10R blockade in P. berghei ANKA-infected BALB/c mice is associated with parasite accumulation in the brain. BALB/c mice were infected i.v. with 10^4^ P. berghei ANKA *luc* pRBCs and either left untreated (control) or treated with anti-IL-10R antibodies. (A) Cumulative survival curve. Closed diamonds (◆), control (*n* = 16); open diamonds (◇), anti-IL-10R antibody (*n* = 15); ***, *P* < 0.0001 (log rank [Mantel-Cox] test). (B) Cumulative incidence of mice developing ECM. ***, *P* < 0.0001 (Fisher's exact test). Similar to Fig. 1, mice that survived were euthanized because of high levels of parasitemia and anemia. (C) Parasitemia levels, shown as the mean ± the standard deviation, of P. berghei ANKA-infected mice. The data shown are representative of two independent experiments performed with five mice per group. *, *P* < 0.05 (Mann-Whitney U test). (D to G) Parasite accumulation in the whole body (D), head (E), and isolated brain (F, G) as measured by luciferase activity on day 7 postinfection. The data shown are representative of two independent experiments performed with five mice per group. In panels D, E, and G, the data shown are the mean ± the standard deviation. *, *P* < 0.05; **, *P* < 0.001 (Mann-Whitney U test).

A key feature of human CM and ECM in mice is the accumulation of pRBCs in the brain microvasculature. To determine the effects of IL-10R signaling on parasite accumulation, control and anti-IL-10R antibody-treated mice were infected with P. berghei ANKA *luc* parasites that express luciferase. The survival curve and cumulative ECM incidence (Fig. 2A and B) of P. berghei ANKA *luc*-infected control and anti-IL-10R antibody-treated mice were similar to those of P. berghei ANKA-infected mice, although the onset of ECM was somewhat delayed. Anti-IL-10R antibody-treated P. berghei ANKA *luc*-infected mice developed ECM between days 7 and 12 postinfection, and control mice survived until day 25. P. berghei ANKA *luc*-infected anti-IL-10R antibody-treated mice presented with significantly lower parasitemia values on days 5 and 7 postinfection than control mice (Fig. 2C). However, when parasite accumulation was measured by luminescence in the whole body (Fig. 2D), head (Fig. 2E), and isolated brain (Fig. 2F and G), the values were significantly higher in anti-IL-10R antibody-treated mice than in control mice. These results indicate that despite a lighter peripheral parasite burden in anti-IL-10R antibody-treated mice than in control mice, the generalized parasite biomass is significantly elevated when the IL-10R signaling pathway is blocked.

**FIG 3:**
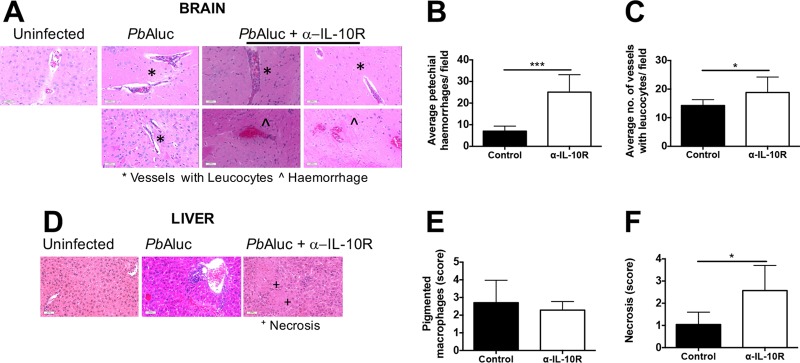
ECM after IL-10R blockade in P. berghei ANKA-infected BALB/c mice is associated with brain pathology. (A) Histological examination of H&E-stained brain sections from uninfected and day 7 P. berghei ANKA (*Pb*A) *luc* pRBC-infected mice (*n* = 7 per group). Asterisks indicate areas of vessels with leukocytes, while circumflexes signify hemorrhages. Magnification, ×20. Graphs show the quantification of the number of petechial hemorrhages (B) and vessels with leukocytes (C). (D) Similar to panel A but with liver sections. Graphs show the scoring of pigmented macrophages (E) and necrosis (F). In panels B, C, E, and F, the data shown are the mean ± the standard deviation. *, *P* < 0.05; ***, *P* < 0.0001 (Mann-Whitney U test).

Histological examination of the brains and livers of P. berghei ANKA *luc*-infected control and anti-IL-10R antibody-treated mice was also performed. The numbers of brain petechial hemorrhages were significantly higher in anti-IL-10R antibody-treated mice than in control mice (Fig. 3A, B), while the numbers of microvessels with leukocytes did not differ between the two groups (Fig. 3C). Moreover, there was also no difference between the numbers of perivascular infiltrates in the liver in the two groups (Fig. 3E), although there was a trend of finding necrosis in anti-IL-10R antibody-treated mice but not in control mice (Fig. 3D and F). Taken together, these findings signify that blocking IL-10R signaling in otherwise ECM-resistant mice results in the development of immune pathology with all of the features of ECM.

### Effector responses are amplified following IL-10R blockade.

**FIG 4:**
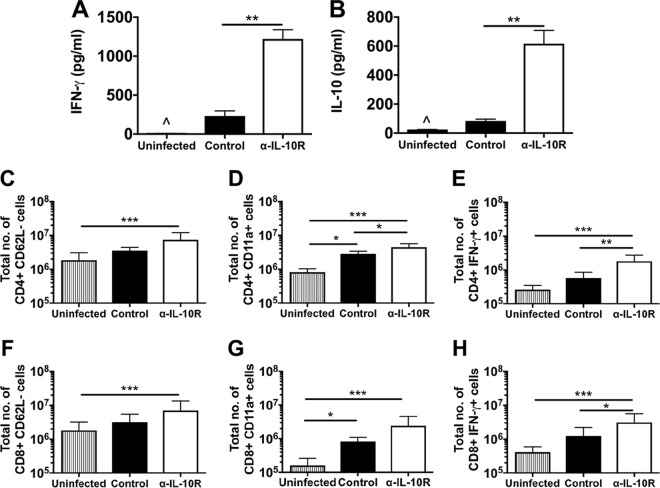
Enhanced effector responses after IL-10R blockade in P. berghei ANKA-infected BALB/c mice. BALB/c mice were infected i.v. with 10^4^ P. berghei ANKA *luc* pRBCs (A and B) or 10^4^ P. berghei ANKA pRBCs (C to E) and either left untreated (control) or treated with anti-IL-10R antibodies. Serum IFN-γ (A) and IL-10 (B) levels were determined with Quantikine kits from R&D Systems. For cellular analyses, splenocytes were prepared from uninfected or day 6 infected mice and stained for surface CD3, CD4, CD8, CD62L, and CD11a. The data shown are gated on CD3^+^ T cells (see Fig. S1). The absolute numbers (mean ± standard deviation) of CD4^+^ CD62L^−^ cells (C), CD4^+^ CD11a^+^ cells (D), CD8^+^ CD62L^−^ cells (F), and CD8^+^ CD11a^+^ cells (G) are shown. Splenocytes were also stimulated with PMA/ionomycin for 5 h in the presence of brefeldin A; this was followed by intracellular IFN-γ staining. The absolute numbers (mean ± standard deviation) of CD4^+^ IFN-γ^+^ cells (E) and CD8^+^ IFN-γ^+^ cells (H) are shown. The results shown are pooled data from two similar experiments (three to five mice per group). The data shown are the mean ± the standard deviation. *, *P* < 0.05; **, *P* < 0.001; ***, *P* < 0.0001 (Kruskal-Wallis test/Dunn's multiple-comparison test).

ECM is a result of T-cell-mediated inflammation in C57BL/6 mice. The development of ECM in P. berghei ANKA-infected anti-IL-10R antibody-treated BALB/c mice was indicative of an amplified inflammatory response. As an indicator of systemic inflammation, serum IFN-γ concentrations (Fig. 4A) were significantly higher in anti-IL-10R antibody-treated mice than in control and uninfected mice on day 7 postinfection. Consistent with the blockade of IL-10R signaling, serum IL-10 concentrations (Fig. 4B) were also significantly higher in anti-IL-10R antibody-treated mice than in control and uninfected mice. The levels of splenic activation were also evaluated on day 6 postinfection. The flow cytometry gating strategies used are shown in Fig. S1A, B, and F in the supplemental material. There was a trend toward larger proportions of splenic CD4^+^ and CD8^+^ T cells expressing CD62L^−^ and CD11a^+^ and producing IFN-γ in the anti-IL-10R antibody-treated mice than in the control and uninfected mice (see Fig. S1B to I). Considering splenic leukocyte counts, a statistically significant difference between the control and anti-IL-10R antibody-treated groups was reached for the total numbers of CD4^+^ T cells expressing CD11a^+^, and CD4^+^, and CD8^+^ T cells producing IFN-γ (Fig. 4C to H). Taken together, the results show that the development of ECM in P. berghei ANKA-infected anti-IL-10R antibody-treated BALB/c mice correlates with higher levels of systemic inflammation and T cell activation.

### Development of ECM in BALB/c mice following IL-10R blockade is mediated by T cells and IFN-γ.

**FIG 5:**
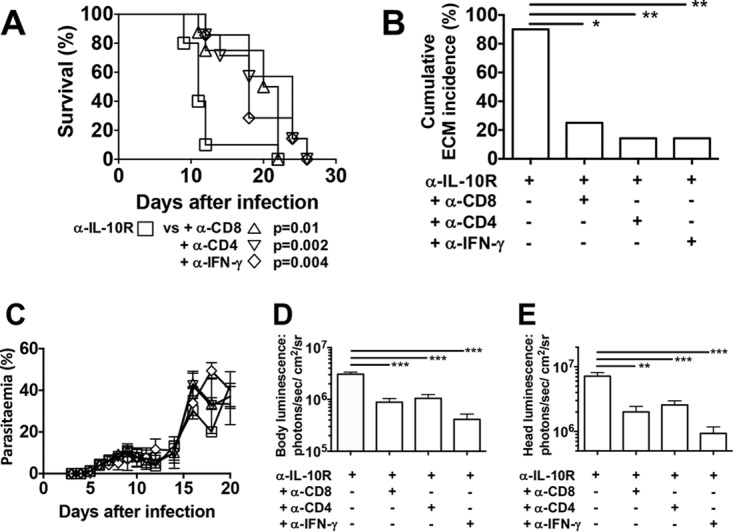
ECM after IL-10R blockade in P. berghei ANKA-infected BALB/c mice is dependent on T cells and IFN-γ. BALB/c mice were infected i.v. with 10^4^ P. berghei ANKA *luc* pRBCs and treated with anti-IL-10R antibody. (A) Cumulative survival curves of IL-10R blockade alone (□, *n* = 10), with anti-CD8^+^ T cell depletion (△, *n* = 8), with anti-CD4^+^ T cell depletion (▽, *n* = 7), and with anti-IFN-γ neutralization (◇, *n* = 7). *P* values (log rank [Mantel-Cox] test) are shown. (B) Cumulative incidence of mice developing ECM. *, *P* < 0.05; **, *P* < 0.001; ***, *P* < 0.0001 (Fisher's exact test). Similar to Fig. 1 and 2, mice that survived were euthanized because of high levels of parasitemia and anemia. (C) Parasitemia levels, shown as the mean ± the standard deviation, of P. berghei ANKA *luc*-infected mice represented by the same symbols as in panel A. (D, E) Parasite accumulation in the whole body (D) and head (E) as measured by luciferase activity on day 7 postinfection. The data shown are the mean ± the standard deviation. **, *P* < 0.001; ***, *P* < 0.0001 (Kruskal-Wallis test/Dunn's multiple-comparison test). The data shown are representative of two independent experiments of at least seven animals per group.

The data presented so far indicate that blockade of IL-10R signaling leads to full ECM susceptibility in otherwise resistant BALB/c mice, and this is associated with amplified T cell activation and higher serum IFN-γ concentrations. These findings are consistent with the hypothesis that signaling through the IL-10R switches off T cell reactivity and thus inhibits the development of ECM. To ascertain whether T cell populations are the targets of IL-10R-mediated regulation, anti-IL-10R antibodies were combined with depletion of antibodies specific for CD4^+^ or CD8^+^ T cells *in vivo*. Depletion of either CD4^+^ or CD8^+^ T cells during P. berghei ANKA *luc* infection prevented the development of ECM in anti-IL-10R antibody-treated mice (Fig. 5A and B). CD4^+^ or CD8^+^ T-cell-depleted mice developed severe anemia and eventually died 2 to 3 weeks postinfection. The role of IFN-γ in the development of ECM in anti-IL-10R antibody-treated P. berghei ANKA *luc*-infected BALB/c mice was also determined by combination with anti-IFN-γ neutralizing antibodies throughout infection. Neutralization of IFN-γ during P. berghei ANKA *luc* infection prevented the development of ECM in anti-IL-10R antibody-treated mice (Fig. 5A and B). P. berghei ANKA *luc*-infected anti-IL-10R antibody-treated mice presented peripheral parasitemia levels similar to those of mice given anti-CD8^+^, -CD4^+^, or -IFN-γ antibodies (Fig. 5C). Interestingly, treatment with anti-CD8^+^, -CD4^+^, or -IFN-γ antibodies led to significantly less parasite accumulation in the whole bodies (Fig. 5D) and heads of infected animals (Fig. 5E). These data demonstrated that while the parasite biomass is increased by IL-10R blockade, the effect is reversed in the absence of T cells or IFN-γ.

## DISCUSSION

Our findings provide an increased understanding of the regulatory pathways that impact the outcome of infection with an ECM-inducing rodent malaria strain that mimics the pathological processes associated with CM due to P. falciparum in humans. We have shown that blocking IL-10R facilitates the development of acute immune pathology in normally ECM-resistant BALB/c mice with pathological features compatible with ECM in susceptible C57BL/6 mice. Neuropathological signs were associated with heavy parasite loads, the development of IFN-γ-secreting CD4^+^ and CD8^+^ T cells in the spleen, and an influx of CD8^+^ T cells into the brains of IL-10R antibody-treated mice. Thus, IL-10R signaling plays a vital role in the prevention of immune-mediated neuropathology during P. berghei ANKA infection of ECM-resistant BALB/c mice.

The outcomes reported in this study expand those reported in our previous work, where blockade of CTLA-4 or PD-1/PD-L1 inhibitory pathways in P. berghei ANKA-infected BALB/c mice also rendered the animals susceptible to ECM. Both studies show that depletion of T cells and neutralization of IFN-γ abrogated the effects of regulatory pathway blockade, validating the notion that ECM has similar etiologies in both BALB/c mice and susceptible C57BL/6 mice. Both studies further corroborate that CD8^+^ T cells and IFN-γ are the critical effectors of ECM. While damage to brain vessels has implicated perforin and granzyme secretion in P. berghei ANKA-infected C57BL/6 mice (12–14), the roles of these molecules in BALB/c mice warrant further investigation. While it appears that the CTLA-4, PD-1/PD-L1, and IL-10-R pathways independently regulate host resistance to P. berghei ANKA infection of BALB/c mice, further studies are also needed to understand the role that the CTLA-4 and/or PD-1/PD-L1 axes are playing in the context of IL-10R and to identify whether there is a primary regulatory pathway for T cells in this infection model.

While our depletion experiments suggest that the pathological effects of IL-10R blockade result from the regulation of T cell responses, additional studies are required to ascertain the roles of IL-10R expression and IL-10R blockade in other immune cells. DCs and monocytes have been shown to produce IL-10 during malaria infections (23–25). Similarly, B cells have also been reported to produce IL-10 in P. berghei ANKA-infected C57BL/6 mice (26). Recently, tissue-resident CD169^+^ macrophages were shown to produce high levels of IL-10 during P. berghei ANKA infection in BALB/c mice and to restrict inflammatory responses (27). Concerning T cells, protection against ECM may also be due to balanced IFN-γ/IL-10 secretion in activated T cells versus regulatory T cells (Tregs). The failure of IL-10 to counterbalance IFN-γ release during IL-10R blockade may be attributed to a failure in the IL-10 signaling pathway in both activated T cells and Tregs with consequent release of IFN-γ. This assumption on Tregs is backed up by recent findings of a loss of FoxP3 expression in Treg cells with increased proinflammatory cytokine release during P. chabaudi AS infection in IL-10R-treated DBA/2 mice (28). Failure of the IL-10R signaling pathway in Tregs has also been associated with loss of FoxP3 expression and inflammatory cytokine release in a murine model of inflammatory bowel disease (29, 30). Furthermore, in the murine model of hepatitis C virus infection, IL-10R blockade steers the inflammatory response toward a type 1 IFN-γ-mediated T cell response (31).

IL-10 production is also increased during P. berghei ANKA infection of ECM-susceptible C57BL/6 mice (32, 33). It is likely that IL-10 performs a similar regulatory role in C57BL/6 mice, although its failure to provide protection against ECM remains unclear. It is possible that an overexuberant inflammatory response supersedes the physiological levels of regulation facilitated by IL-10R signaling. Alternatively, downstream IL-10R signaling is impaired on target cells.

Although the kinetics and precise interactions between the effector and regulatory pathways remain undefined in this model of ECM, our studies reveal the importance of maintaining a balance between effector and regulatory immune responses for preventing ECM. Disproportionate proinflammatory responses consistently lead to immunopathology, although there is reasonable control of parasitemia. Equally, poor antiparasite immune responses generated concurrently with strong regulatory responses permit parasitemia with harmful consequences, as demonstrated in this P. berghei ANKA/BALB/c model of severe malaria.

## MATERIALS AND METHODS

### Animals.

In the United Kingdom, 6- to 12-week-old female BALB/cAnNCrl mice were purchased from Charles Rivers UK Ltd. and maintained under barrier conditions. Animal experiments were approved by the London School of Hygiene and Tropical Medicine Animal Welfare and Ethical Review Board and performed under Animals (Scientific Procedures) Act 1986. In Singapore, 6- to 7-week-old female BALB/cJ mice were bred and kept under specific-pathogen-free conditions in the Biomedical Resource Center of A*STAR. Animal experiments were approved by the A*STAR Institutional Animal Care and Use Committee in accordance with the rules and regulations of the Singaporean Agri-Food and Veterinary Authority and the National Advisory Committee for Laboratory Animal Research.

### Parasites and experimental infections.

Experimental infections were initiated by intravenous (i.v.) inoculation of 10^4^ pRBCs. In the United Kingdom, P. berghei ANKA parasites (P. berghei ANKA clone 15cy1 [34], referred to here simply as P. berghei ANKA) expressing green fluorescent protein were used. In Singapore, transgenic parasite P. berghei ANKA line 231cl1 expressing luciferase and green fluorescent protein (referred to here as P. berghei ANKA *luc*) was provided by Christian Engwerda (QIMR Berghofer Medical Research Institute, Brisbane, Australia). Infected mice were monitored for neurological signs (uncoordinated and reduced locomotion, paralysis, deviation of the head, ataxia, convulsions, and coma). Parasitemia levels were monitored by the examination of Giemsa-stained thin blood smears or by flow cytometry (35). In some experiments, day 7 infected mice were sacrificed and perfused and their brains and livers were removed for histology or imaging (see below). Serum was stored at −70°C for cytokine quantification (see below).

### *In vivo* administration of antibodies.

All antibodies were administered by intraperitoneal injection. Blocking antibody to IL-10R [1B1.3A] was administered at 0.3 mg/mouse on day −1 postinfection and at 0.2 mg/mouse on days 1, 4, and 6 postinfection. Neutralizing antibody to IFN-γ [XMG1.2] was administered at 0.4 mg/mouse on days 4 and 6 postinfection, and depleting antibodies to CD4 [GK1.5] and CD8 [53.6.72] were administered at 1 mg/mouse on day 6 postinfection. All antibodies were rat anti-mouse IgG, obtained from BioXCell (USA); control rat IgG was obtained from Pierce.

### Flow cytometry.

The antibodies (clones) used for cell surface staining were (obtained from eBioscience) anti-mouse CD4 (GK1.5), CD8 (53.6-7), CD11a (M17/4), and CD62L (MEL-14) or (obtained from BD Biosciences) anti-mouse CD3 (145-2C11), CD4 (RM4-5), and CD8 (53-6.7). Isolated brain and splenic leukocytes were directly stained in accordance with standard protocols (15). Antibodies used for intracellular staining were obtained from eBioscience (IFN-γ [XMG1.2]). Intracellular staining was performed by permeabilizing cells with 0.1% saponin–PBS. Cells were analyzed with a FACSCalibur or LSR II (BD Immunocytometry Systems) and FlowJo software (TreeStar). Gating strategies were performed as previously described, where T cells are gated from singlets/live^+^ and lymphocyte populations (15). Absolute numbers of lymphocytes were calculated from cell counts.

### Cytokine quantification.

Serum cytokine levels were assayed with the IFN-γ Quantikine kit and the Quantikine ELISA mouse IL-10 kit (both from R&D Systems) in accordance with the manufacturer's protocol. Intracellular IFN-γ levels were measured by flow cytometry (as described above) following 5 h of spleen cell culturing in the presence of phorbol myristate acetate (PMA; 50 ng/ml), ionomycin (1 μg/ml), and brefeldin A (1 μg/ml).

### Bioluminescent imaging.

Daily *in vivo* imaging was done to monitor the P. berghei ANKA *luc* parasite distribution (IVIS; Xenogen, Alameda, CA). Infected mice were shaved, anesthetized, and injected subcutaneously with 100 μl of d-luciferin potassium salt (5 mg/ml in PBS; Caliper Life Sciences). Two minutes later, bioluminescence images were acquired with a medium binning factor and a field of view (FOV) of 21.7 cm for the whole body (ventral) or 4 cm for the head (dorsal). The exposure imaging time was 5 to 60 s. For *ex vivo* imaging, mice were given a second injection of luciferin and anesthetized and 3 min later perfused and sacrificed. Brains were removed and imaged with a 10-cm FOV. To allow comparisons of images from different days, uninfected mice injected with luciferin were imaged for background subtraction. Bioluminescence quantification was done by using Living Imaging 4.2 software and expressed in average radiance units (photons per second per square centimeter per steradian).

### Histology.

Infected mice were sacrificed on day 7 postinfection. Mice were perfused with PBS, and their isolated brains and livers were immersed in 4% formaldehyde and embedded in paraffin. Sagittal sectioning was performed to obtain 5-μm-thick brain and liver sections, which were stained with hematoxylin and eosin (H&E). Slides were acquired on a Metafer4 (MetaSystems), and numbers of hemorrhages, leukocytes, and necroses (of the liver) were manually determined.

### Statistical analysis.

Differences in survival were assessed with the log rank (Mantel-Cox) test, and Bonferroni correction was used to adjust for multiple comparisons within the log rank (Mantel-Cox) test. The cumulative ECM incidence between two groups was analyzed with Fisher's exact test. Comparisons of two groups were made with the Mann-Whitney test, and for multiple comparisons (more than two groups), statistical significance was determined with the Kruskal-Wallis test with Dunn's posttest. For log-transformed values to allow normal distribution of the data, analysis of variance with Bonferroni's posttest for multiple groups was used. The data were analyzed with GraphPad Prism software (version 6.0) with *P* < 0.05 as the level of significance.

## Supplementary Material

Supplemental material
