# Supplementary material for: Host Resistance to Plasmodium-Induced Acute Immune Pathology Is Regulated by Interleukin-10 Receptor Signaling
Source: Infect Immun. 2017 May 23;85(6):e00941-16. doi: 10.1128/IAI.00941-16 (PMC5442633; doi:10.1128/IAI.00941-16)
Supplement: Supplemental material [file supp_85_6_e00941-16__index.html]

Host Resistance to Plasmodium-Induced Acute Immune Pathology Is Regulated by Interleukin-10 Receptor Signaling — Supplemental material 

# Host Resistance to Plasmodium-Induced Acute Immune Pathology Is Regulated by Interleukin-10 Receptor Signaling

## Supplemental material

- Supplemental file 1 -

  Fig. S1. Flow cytometric analysis of splenocytes after IL-­10R blockade in *Pb*A-­infected BALB/c mice.

  PDF, 119K
