# Supplementary material for: Host Resistance to Plasmodium-Induced Acute Immune Pathology Is Regulated by Interleukin-10 Receptor Signaling
Source: Infect Immun. 2017 May 23;85(6):e00941-16. doi: 10.1128/IAI.00941-16 (PMC5442633; doi:10.1128/IAI.00941-16)
Supplement: Supplemental material [file IAI.00941-16_zii999092059s1.pdf]

Host resistance to *Plasmodium*-induced acute immune pathology is regulated by IL-10 receptor signalling

Carla Claser, J. Brian De Souza, Samuel G. Thorburn, Georges Emile Grau,
Eleanor M. Riley, Laurent Rénia, Julius C. R. Hafalla

Figure S1. Flow cytometric analysis of splenocytes after IL-10R blockade in *Pb*A-infected BALB/c mice.

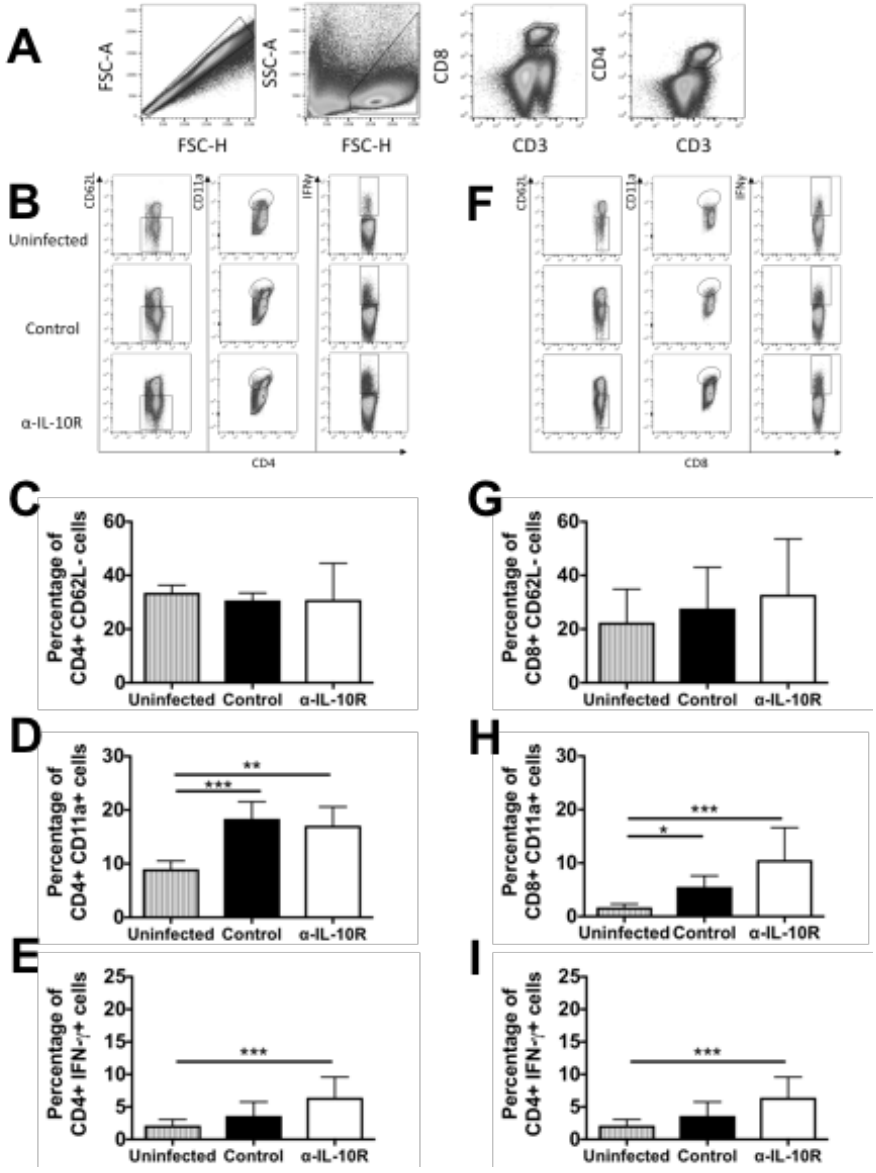

Figure S1. Flow cytometric analysis of splenocytes after IL-10R blockade in *Pb*A-infected BALB/c mice. Methodology is similar to Figure 4 legend. BALB/c mice were infected i.v. with 10^4 *PbA* pRBCs and were either untreated (control) or treated with α-IL-10R antibodies. Splenocytes were prepared from uninfected or day 6 infected mice and stained for surface CD3, CD4, CD8, CD62L and CD11a. Splenocytes were also stimulated with PMA/Ionomycin for 5 hours in the presence of Brefeldin A followed by intracellular IFN-γ staining. **(A)** Gating strategies for singlets, lymphocytes, as well as $CD3^+$ $CD4^+$ and $CD3^+$ $CD8^+$ cells. **(B,F)** Gating strategies for the expression of $CD62L^-$, $CD11a^+$ and production of IFN-γ. The proportions (mean $\pm$ SD) of **(C)** $CD4^+$ $CD62L^-$ cells, **(D)** $CD4^+$ $CD11a^+$ cells, **(G)** $CD8^+$ $CD62L^-$ cells, and **(H)** $CD8^+$ $CD11a^+$ cells are shown. Splenocytes were also stimulated with PMA/Ionomycin for 5 hours in the presence of Brefeldin A followed by intracellular IFN-γ staining. The proportions (mean $\pm$ SD) of **(E)** $CD4^+$ IFN-γ^+ cells and **(I)** $CD8^+$ IFN-γ^+ cells are shown. Results are pooled data from two similar experiments (3-5 mice per group). Data are shown as mean ± SD, * P<0.05, ** P<0.001 and *** P<0.0001 (Kruskal-Wallis Test/Dunn's multiple comparison test).
